# Sodium tanshinone IIA sulfonate protects ARPE-19 cells against oxidative stress by inhibiting autophagy and apoptosis

**DOI:** 10.1038/s41598-018-33552-2

**Published:** 2018-10-11

**Authors:** Dongmei Han, Xingwei Wu, Libin Liu, Wanting Shu, Zhenping Huang

**Affiliations:** 1 Department of Ophthalmology, Jinling Hospital, Nanjing, 210002 China; 2 Department of Ophthalmology, Shanghai General Hospital, Shanghai Jiaotong University School of Medicine, Shanghai, 200080 China; 3 The Third People’s Hospital of Jingdezhen, Jingdezhen, 333000 China

## Abstract

Oxidative stress in retinal pigment epithelium (RPE) is considered to be a major contributor to the development and progression of age-related macular degeneration (AMD). Previous investigations have shown that sodium tanshinone IIA sulfonate (STS) can alleviate oxidative stress in haemorrhagic shock-induced organ damage and cigarette smoke-induced chronic obstructive pulmonary disease in mice. However, whether STS has a protective effect in ARPE-19 cells under oxidative stress and its exact mechanisms have not yet been fully elucidated. In the present study, we utilized H_2_O_2_ to establish an oxidative stress environment. Our findings show that STS activated the PI3K/AKT/mTOR pathway to inhibit autophagy and diminished the expression of the autophagic proteins Beclin 1, ATG3, ATG7 and ATG9 in ARPE-19 cells under oxidative stress. Detection of the intrinsic apoptosis-related factors BAX, mitochondrial membrane potential (MMP), caspase-9, caspase-3 and BCL-2, as well as the extrinsic apoptosis-related factors c-FLIP, v-FLIP and caspase-8, confirmed that STS inhibited the intrinsic and extrinsic apoptotic pathways, and attenuated apoptosis in ARPE-19 cells under oxidative stress conditions. These findings shed new light on the protective effects of STS in ARPE-19 cells and its mechanisms under oxidative stress to provide novel and promising therapeutic strategies for AMD.

## Introduction

Age-related macular degeneration (AMD) is a progressive and devastating neurodegenerative malady that is the leading cause of blindness among the elderly in developed countries. AMD is becoming similarly important in the developing world in association with increasing longevity and the westernization of the diet and lifestyle^1^. Mounting evidence has shown that AMD is involved in the degeneration of retinal pigment epithelium (RPE), photoreceptor cells, and choroidal capillaries, of which the dysfunction and degeneration of RPE is pivotal to AMD pathogenesis. The RPE performs several functions that are essential to maintain normal retinal physiology and visual function including lightenergy adsorption, ion and water transport, immunological barrier formation, visual product recycling, phagocytosis, and secretion of growth factors and cytokines^2^. Consequently, RPE defects and/or atrophy secondary to ageing, injury (traumatic or toxic), and diseases can lead to photoreceptor degeneration and vision loss^3^. In addition to support photoreceptor survival and visual function, the RPE also controls formation and maintenance of the choriocapillaris. Clinical and experimental evidences have indicated that the developmental formation of the choroidal vasculature depends on proper RPE differentiation^4^. It is worth noting that RPE resides in an oxygen rich environment, and RPE mitochondrial DNA (mtDNA) is particularly prone to oxidative damage^5^. Oxidative stress in the RPE is hypothesized to be a major contributor to the onset and development of AMD^6^. The ARPE-19 cell line has been widely used to evaluate RPE function and their hypersensitivity to VEGF action, loss of pigmentation, and weaker tight junctions, are all properties which somewhat resemble the aged eye or pathologic conditions^7^. Therefore, the ARPE-19 cell line was used in our study.

Studies have shown that autophagy plays an indispensable role in the pathogenesis of a variety of diseases, including those involving retinal degenerative diseases, such as AMD. In the majority of cases, the induction of autophagy in response to stress acts as a pro-survival mechanism, however, it is now clearly evident that autophagy has a dual role^8^. This degradative mechanism for long-lived proteins and damaged organelles that occurs via the autophagy–lysosomal pathway can provide the possibility of cellular self-destruction under chronic stress conditions^9^. RPE cells can also be induced to undergo autophagy-associated cell death by starvation and oxidative stress^10^.

Sodium tanshinone IIA sulfonate (STS), a derivative of tanshinone IIA, is a water-soluble pharmacologically active component that has been isolated from the rhizome of the Chinese herb Salvia miltiorrhiza, a well-known traditional Chinese medicine, and is widely used for the treatment of cardiovascular diseases. Recent studies have indicated that the beneficial effects of STS in cardiovascular diseases are attributable to its role in reducing ROS production and decreasing pro-inflammatory cytokines^11,12^. Previous study showed that STS prevent lipopolysaccharide-induced inflammation through suppressing NF-κB signaling pathway in endothelial cells, indicating the potential utility of STS for the treatment of inflammatory diseases^13^. In addition, STS treatment was shown to ameliorate organ dysfunction, reduce oxidative stress, and suppress inflammatory responses, which attenuated hemorrhagic shock-induced activation of the NF-κB pathway in rats^14^. STS inhibited cigarette smoke extract (CSE)-induced inflammation and oxidative stress in macrophages in chronic obstructive pulmonary disease mice and these protective effects of STS are associated with the inhibition of CSE-induced HIF-1a expression^15^. Another mechanistic study revealed that increased JNK phosphorylation stimulated by H_2_O_2_ was abolished by STS treatment in adult mice^16^.

In light of these findings, it is plausible and feasible to investigate whether STS can protect ARPE-19 cells against oxidative stress and the specific mechanisms involved in this process. In the present study, we established an oxidative stress environment based on the half-maximal (50%) inhibitory concentration (IC_50_) of H_2_O_2_ as determined by MTT and CCK8 assays and conducted a series of experiments to investigate the protective effect of STS in ARPE-19 cells and its possible mechanism under oxidative stress. In light of the paramount role of RPE in retinal physiology, pathophysiology and the pathogenesis of AMD, our research could provide a new therapeutic strategy for AMD (both dry and wet) that can slow or cease the occurrence and development of this debilitating disease.

## Results

### H_2_O_2_ treatment inhibits the growth of ARPE-19 cells

**Figure 1:**
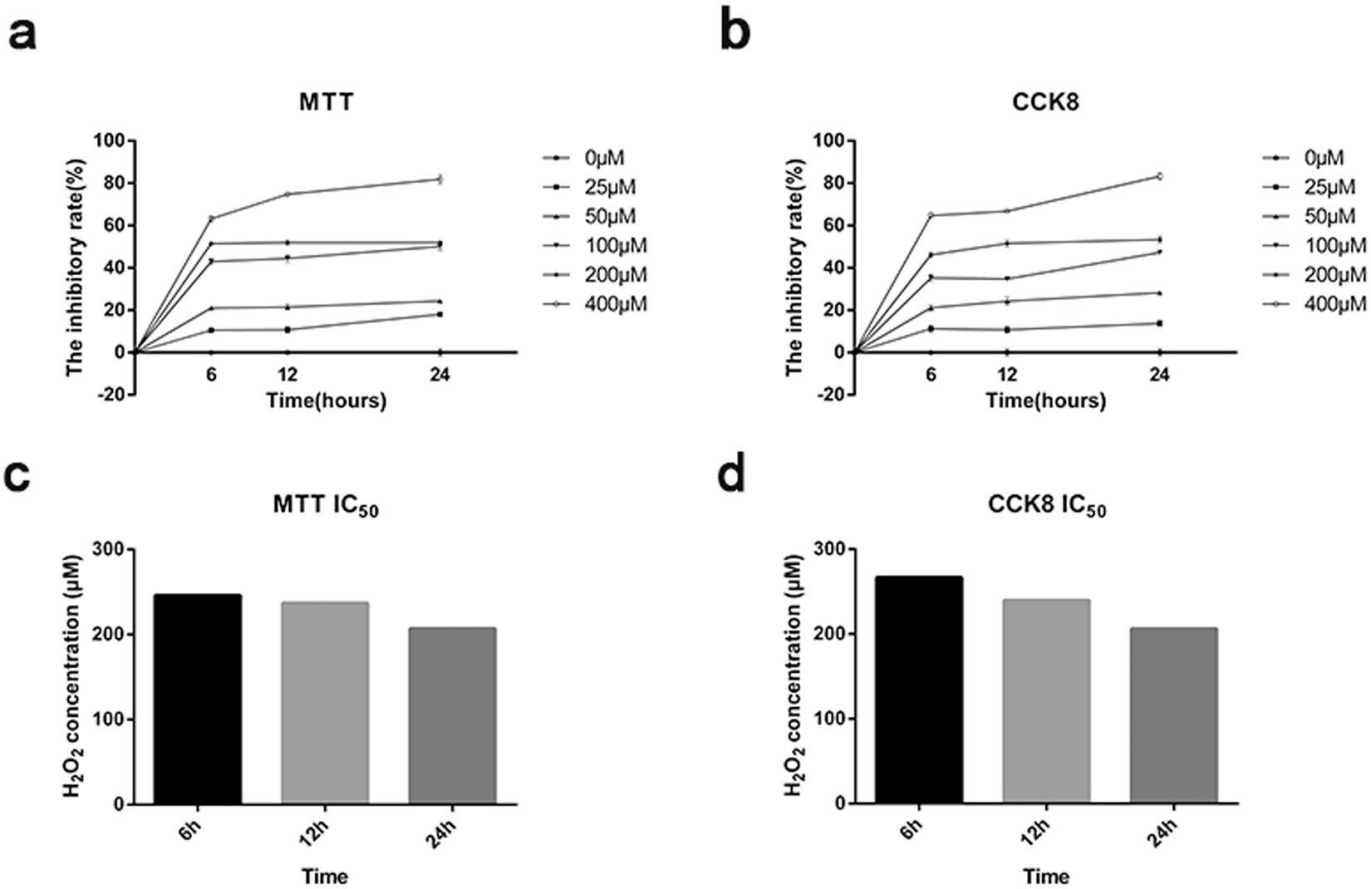
H_2_O_2_ treatment inhibits the growth of ARPE-19 cells in a time- and concentration-dependent manner. (**a**) The results of MTT analysis. (**b**) The results of CCK8 analysis. (**c**) IC_50_ value calculated using MTT assay. (**d**) IC_50_ value calculated using CCK8 assay. Each group of experiments was repeated 3 times, data were analyzed using two-way ANOVA, and the average was taken to draw the line chart and calculate the IC_50_ value at each pointed-time.

We first determined whether H_2_O_2_ treatment could result in ARPE-19 cell growth inhibition using MTT and CCK8 assays. The MTT assay establishes the cytotoxicity of a compound based on decreases in the intracellular NAD(P)-H-dependent oxidoreductase activity^17^. However, the chromogenic product of MTT is insoluble and must, therefore, be solubilized prior to spectrophotometric analysis^18^. To avoid this step, several functionally identical tetrazolium dyes that produce soluble formazans, such as CCK8, have been developed^19^. As shown in Fig. 1a,b, the results of the MTT and CCK8 analysis both showed that the growth inhibition rate of ARPE-19 cells was significantly increased with an escalating H_2_O_2_ concentration and stimulation time. Notably, both the MTT and CCK8 assay results showed that the growth inhibition rate of ARPE-19 cells was approximately 50% at 200 μM H_2_O_2_ and stimulation times of 6 h, 12 h and 24 h, indicating that at this concentration the cell growth inhibition is relatively stable.

### The determination of IC_50_ value

Determination of the half-maximal (50%) inhibitory concentration (IC_50_) is essential for understanding the pharmacological and biological characteristics of a chemotherapeutic agent^20^. The concentration corresponding to a survival rate of 50% is defined as the IC_50_^21^. Currently, MTT assay is widely used for the determination of IC_50_ value. To avoid the unavoidable error of the IC value measured by MTT assay alone, CCK8 assay also used to calculate the IC_50_ value. IC_50_ values at each pointed-time were calculated using the formula: lgIC50 = Xm-I (P- (3-Pm-Pn) / 4). As shown in Fig. 1c,d, the IC_50_ values calculated using the results of MTT and CCK8 assay were also nearest to 200 μM when H_2_O_2_ was added to ARPE-19 cells for 24 hours. Combined with the cellular growth inhibition rate revealed by MTT and CCK8 assay, the IC_50_ value was determined to 200 μM of H_2_O_2_, and simulation time of 24 h. This result was carried out in the subsequent series of experiments.

### H_2_O_2_ treatment increases autophagy in ARPE-19 cells

**Figure 2:**
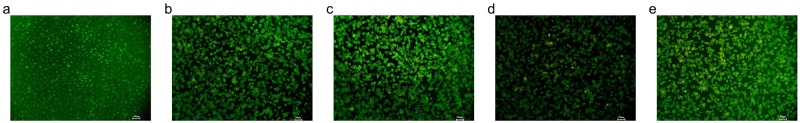
AO staining for autophagosome formation in ARPE-19 cells after cells were treated with escalating concentrations of H_2_O_2_ for 24 hours. (**a**) Untreated cells, (**b**) Cells treated with 100 μM H_2_O_2_. (**c**) Cells treated with 200 μM H_2_O_2_. (**d**) Cells treated with 300 μM H_2_O_2_. (**e**) Cells treated with 400 μM H_2_O_2_. Each group of experiments was repeated 3 times and a representative photo is shown. The images were photographed using 10× objects.

Although a number of studies have shown that autophagy induced in ARPE-19 cells protects the cells in early oxidative stress conditions, it is clear that cell death occurs when excessive oxidative stress exceeds the antioxidant capacity of the cells, resulting in autophagic cell death. Therefore, we first determined whether H_2_O_2_ treatment could induce autophagy in ARPE-19 cells. The formation of autophagosomes is a prominent hallmark of autophagy. In the current study, the promotion of autophagy in ARPE-19 cells challenged by H_2_O_2_ was validated based on acridine orange (AO) staining of autophagosomes^22^. ARPE-19 cells were challenged by 100 μM, 200 μM, 300 μM and 400 μM H_2_O_2_ for 24 h, and untreated cells served as controls. Consistent with our hypothesis, as shown in Fig. 2, red-yellow acidic bodies indicating the formation of autophagosomes appeared after ARPE-19 cells were treated with 100 μM H_2_O_2_ for 24 h. The density of the acidic bodies significantly increased with escalating H_2_O_2_ concentrations, which confirmed that H_2_O_2_ treatment could induce autophagy in ARPE-19 cells, and did this in a concentration-dependent manner when the exposure treatment was fixed.

### STS inhibits autophagic flux in ARPE-19 cells challenged by H_2_O_2_

Next, we made an attempt to assess whether STS could affect H_2_O_2_-induced autophagy in ARPE-19 cells. Because the mean lifespan of an autophagosome is approximately 10 min, the number of autophagosomes at a specific time point does not provide useful information about autophagy per se, as the autophagosome levels can also be increased, for example, during lysosomal dysfunction^23^. Therefore, the detection of autophagosomes alone can not completely represent the autophagy process, however, this obstacle can be circumvented by determining the dynamic process of autophagy that is autophagic flux. Accumulation of autophagosomes may be associated either with an increase of autophagosome synthesis or a disruption of autophagosome–lysosome fusion, or both. To distinguish these two possible pathways, we measured the autophagic flux in the presence of autophagy inhibitors. In this study, 5 mM of the autophagy inhibitor 3MA was utilized in the subsequent series of experiments according to the manufacturer’s protocol.

**Figure 3:**
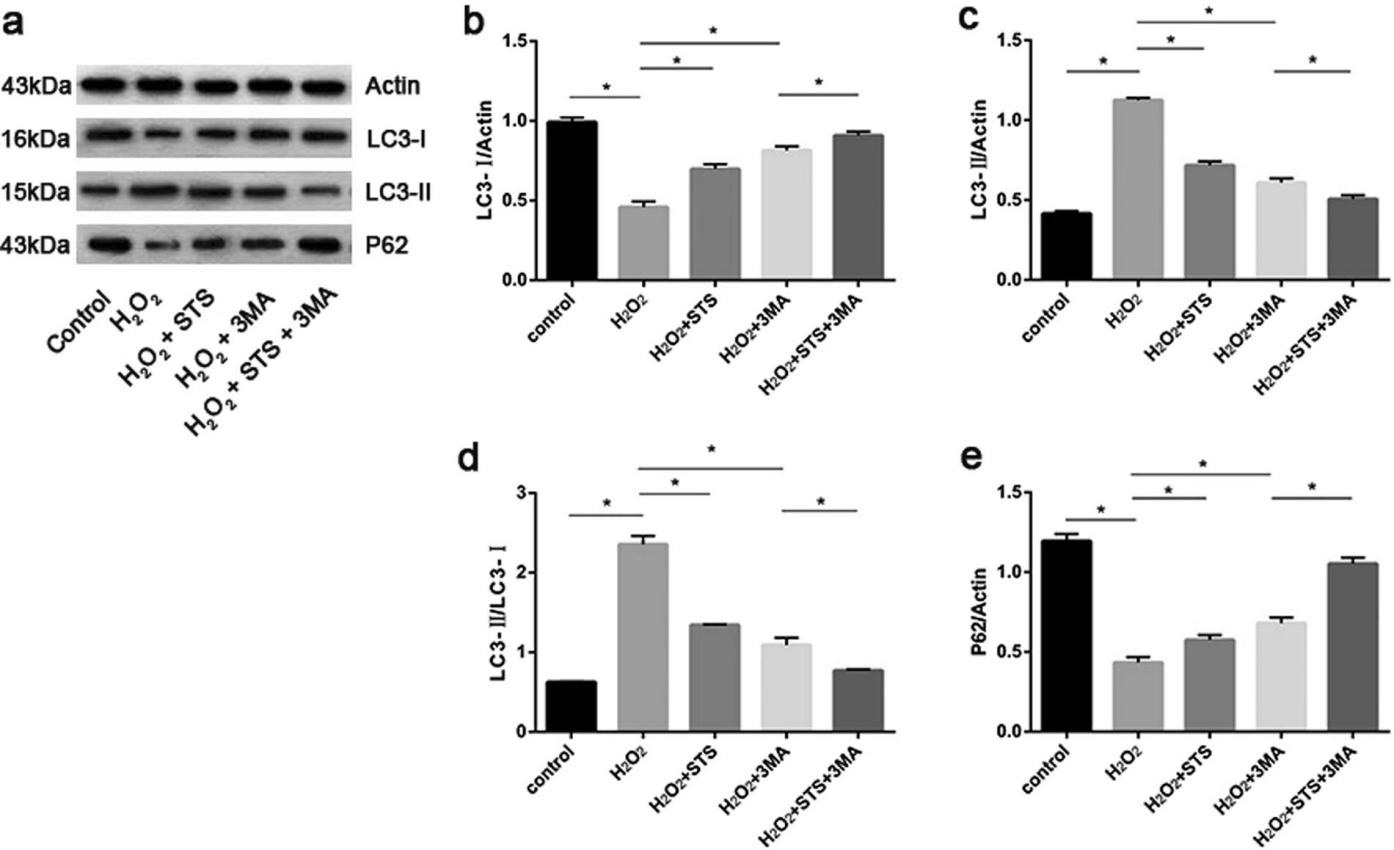
STS inhibits autophagy in ARPE-19 cells under oxidative stress. (**a**) The protein expression levels of LC3-I, LC3-II and p62 were detected by WB and representative protein gel blots are shown. Actin was utilized as a loading control. (**b**–**e**) The accompanied histogram of densitometric quantification of LC3-I, LC3-II, LC3-II/-I and p62. Untreated cells were used as a positive control. Experiments in each group were repeated 3 times. Data were analyzed using one-way ANOVA and differences between means were considered statistically significant when P < 0.05, Bonferroni correction of P-value was applied in multiple comparison. The statistical results showed that the comparison among groups was statistically significant.

LC3-I, a cytosolic protein, undergoes lipidation to form LC3-II (the membrane-bound lipidated form), which is involved in the formation of autophagosome^24^. Therefore, an increased amount of the smaller-molecular-weight LC3-II protein and the ratio of LC3-II to LC3-I is extensively regarded as an essential hallmark of autophagic activation and is linked with an increased number of autophagosomes. Western blot (WB) analysis with anti-LC3 antibody, as shown in Fig. 3a–d and Supplementary Fig. S1, revealed that the expression of LC3-I was significantly decreased in ARPE-19 cells treated with H_2_O_2_ for 24 h, and the expression of LC3-II and the LC3-II/-I ratio were distinctly increased in cells under the same conditions. It is noteworthy that both the expression of LC3-I was markedly increased and the expression of LC3-II and the LC3-II/-I ratio were significantly decreased in ARPE-19 cells treated with STS.

To further confirm the inhibitory effect of STS on autophagy induced by H_2_O_2_ in ARPE-19 cells, we monitored the protein expression of p62 by WB. Also known sequestosome 1 (SQSTM1), p62 possesses a short LC3 interaction region that facilitates direct interaction with LC3 and causes p62 degradation by autophagy^25^. As p62 degradation depends on autophagy, the level of p62 protein is another indicator of autophagic flux^26^. Therefore, a reduction of p62 as evidenced by WB in H_2_O_2_-treated cells would support an increased autophagic degradation. As shown in Fig. 3a,e and Supplementary Fig. S1, a significant reduction in p62 expression was shown in ARPE-19 cells treated with H_2_O_2_, whereas a remarkable elevation in its expression was exhibited in cells treated with STS under oxidative stress.

As mentioned above, these results demonstrate that a prominent increase in autophagic flux occurs in ARPE-19 cells that are treated with H_2_O_2_, whereas STS intervention significantly decreased this flux under oxidative stress. The results of the 3MA intervention demonstrated that the autophagic process occurring in ARPE-19 cells under oxidative stress involves autophagosome formation rather than the disruption of autophagy-lysosomal fusion.

### STS reduces the expression of key autophagic proteins in ARPE-19 cells challenged by H_2_O_2_

**Figure 4:**
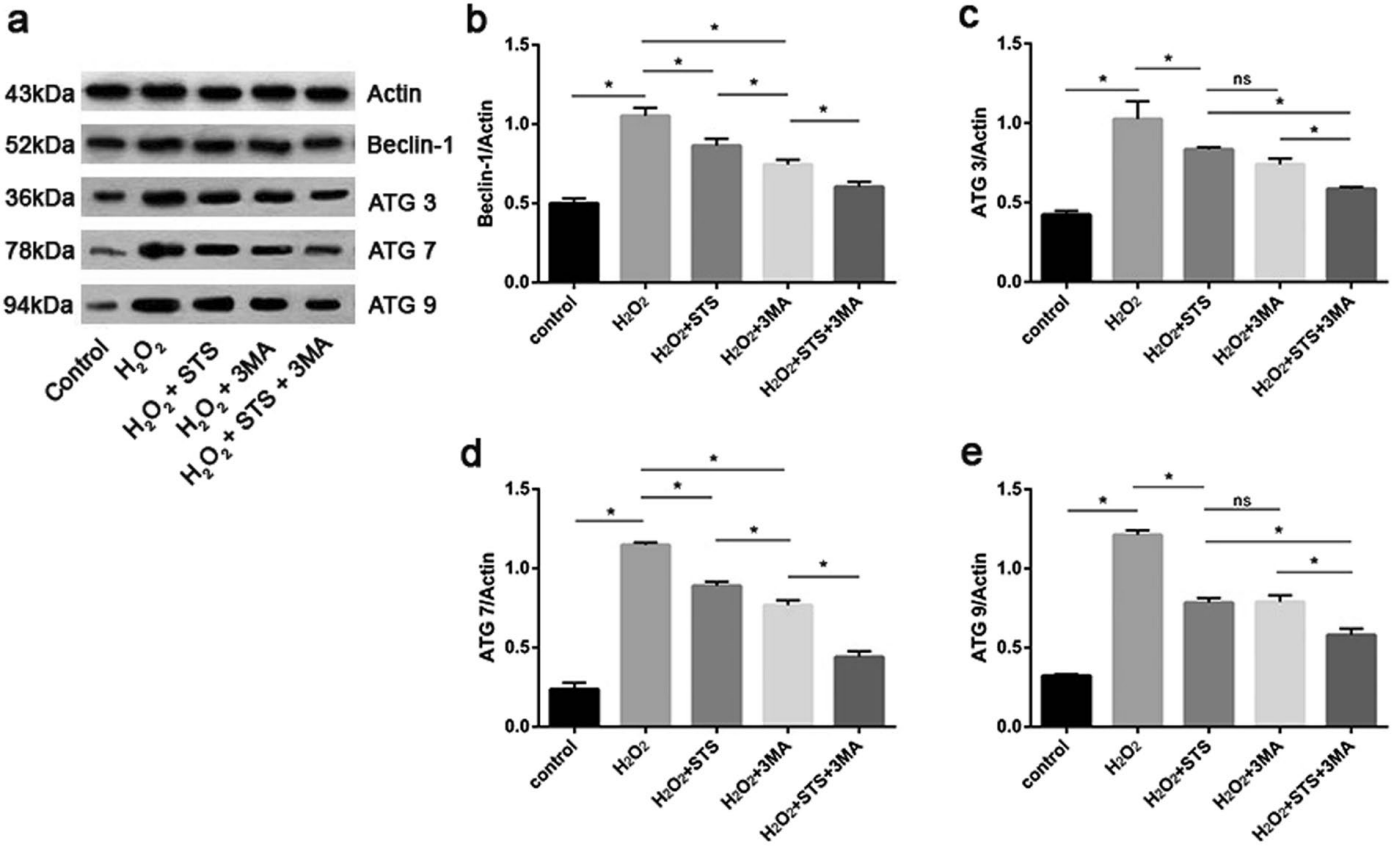
STS decreases autophagic protein expression in ARPE-19 cells under oxidative stress. (**a**) Representative protein gel blots of Beclin 1, ATG3, ATG7 and ATG9 are shown. Actin was utilized as a loading control. (**b**–**e**) The accompanied histogram of densitometric quantification of Beclin1, ATG3, ATG7 and ATG9. Untreated cells were used as positive control. Experiments in each group were repeated 3 times. Data were analyzed using one- way ANOVA, and differences between means were considered statistically significant when P < 0.05, Bonferroni correction of P-value was applied in multiple comparison. Statistical analysis showed that there was no significant difference between H_2_O_2_ + STS and H_2_O_2_ + 3MA in C and E, and the other groups showed statistically significant differences.

All stages of the autophagy process, from the formation of autophagosomes to the fusion of autophagosomes and lysosomes, are regulated by the ATG protein family. To date, over 35 ATG genes have been identified and are shown to finely orchestrate the process of autophagy^27^. To further enucleate the mechanism by which STS inhibits increased autophagy in ARPE-19 cells under oxidative stress, we detected the expression of the key autophagic proteins, Beclin1, ATG3, ATG7 and ATG9, by WB. As shown in Fig. 4a–e and Supplementary Fig. S2, a conspicuous increase in the expression of these four autophagy-related proteins was shown in ARPE-19 cells treated with H_2_O_2_, indicating that H_2_O_2_ treatment increases autophagy in cells by elevating the expression of key autophagic proteins. STS intervention, however, notably diminished the protein expression of Beclin1, ATG3, ATG7 and ATG9 under oxidative stress, indicating that STS inhibits autophagy by inhibiting the expression of these key autophagic proteins in ARPE-19 cells. In addition, the increased expression levels of Beclin 1, Atg3, Atg7 and Atg9 by H_2_O_2_ treatments were partially inhibited by STS intervention and seemed more effectively inhibited by the co-treatment with 3MA.

### STS inhibits autophagy induced by H_2_O_2_ in ARPE-19 cells through activating the PI3K/AKT/mTOR signaling pathway

**Figure 5:**
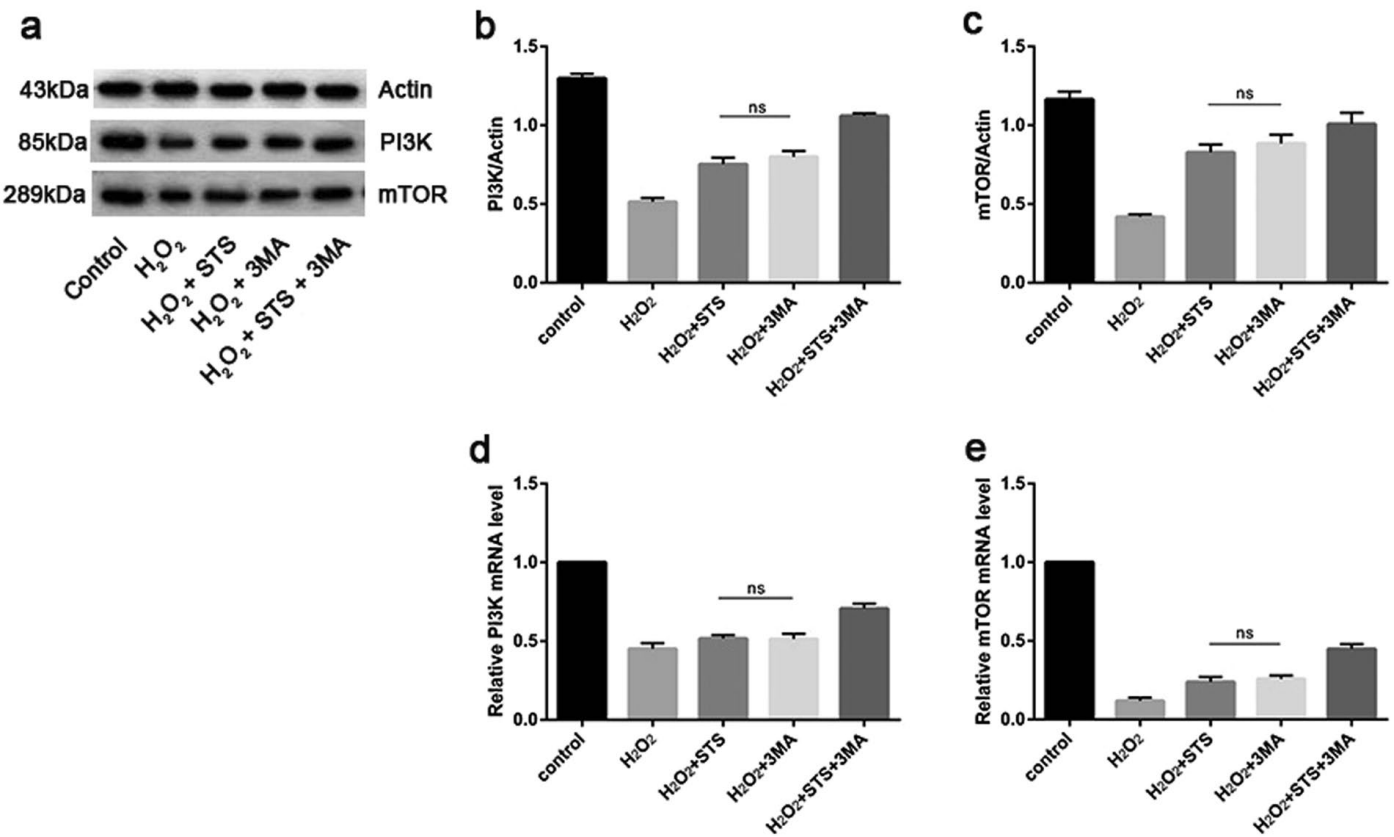
STS inhibits autophagy by activating the PI3K/AKT/mTOR pathway in ARPE-19 cells under oxidative stress. (**a**) Representative protein gel blots of PI3K and mTOR are shown for ARPE-19 cells. Actin was utilized as a loading control. The experiment was repeated 3 times in each group. (**b**–**c**) The accompanied histogram of densitometric quantification of PI3K and mTOR. Untreated cells were used as a positive control. (**d**–**e**) RT-qPCR analysis of mRNA levels of PI3K and mTOR in ARPE-19 cells. Actin was used as an internal control for the experiment. The mRNA levels were normalized to the actin level. Bars represent the mean ± SD of six independent experiments. Data were analyzed using one-way ANOVA and differences between means were considered statistically significant when P < 0.05, Bonferroni correction of P-value was applied in multiple comparison. There was no statistical difference between H_2_O_2_ + STS and H_2_O_2_ + 3MA in (**b**–**d**).

To clarify the molecular mechanism by which STS inhibits increased autophagy in ARPE-19 cells under oxidative stress, we analyzed possible pathways involved in this process. The mTOR kinase-dependent signaling pathway controls autophagy^28^. Activation of the phosphoinositide PI3K/AKT/mTOR pathway inhibits autophagy, whereas the loss of signaling through this cascade removes the negative repression of mTOR^28^. First, we detected the expression of PI3K and mTOR by WB. As shown in Fig. 5a–c and Supplementary Fig. S3, H_2_O_2_ treatment greatly diminished the protein expression levels of PI3K and mTOR in ARPE-19 cells in comparison with untreated cells, indicating that H_2_O_2_ activates autophagy by inactivating the PI3K/AKT/mTOR pathway in ARPE-19. In contrast, STS treatment strikingly increased the expression of PI3K and mTOR, confirming that STS inhibits autophagy by activating the PI3K/AKT/mTOR pathway under oxidative stress. To investigate whether the STS inhibition of ARPE-19 cells autophagy under oxidative stress occurs at the mRNA level, RT-qPCR was utilized to assess mRNA expression in each group. As shown in Fig. 5d,e and Supplementary Fig. S3, and consistent with our supposition, a significant decrease was seen for the PI3K mRNA and mTOR mRNA levels in ARPE-19 cells challenged by H_2_O_2_, indicating that H_2_O_2_ inactivates PI3K/AKT/mTOR pathway and then activates autophagy by inhibiting PI3K and mTOR transcription. In contrast, a dramatic increase of PI3K and mTOR mRNA levels was found in cells treated with STS under oxidative stress, indicating that the STS could increase PI3K and mTOR transcription, activate the PI3K/AKT/mTOR pathway, and lessen autophagy.

### H_2_O_2_ treatment induces apoptosis in ARPE-19 cells

**Figure 6:**
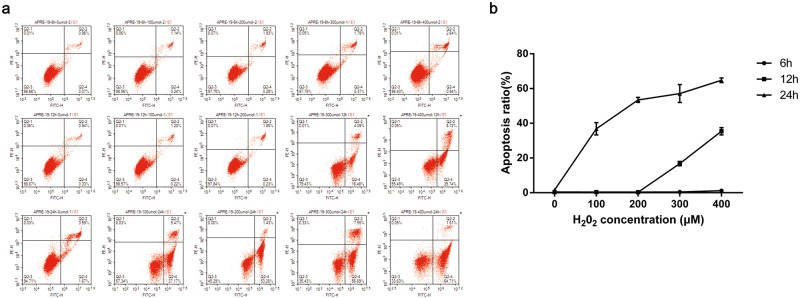
H_2_O_2_ treatment results in apoptosis in a time- and concentration-dependent manner in ARPE-19 cells. (**a**) Typical flow cytometry images of apoptosis in each group using V-FITC/PI labelling. (**b**) The accompanied line chart. Each group of experiments repeated 3 times and the average was applied to produce the line chart, data were analyzed using two-way ANOVA.

In general, apoptosis is the final stage of various programmed cell death patterns (apoptosis, autophagic cell death and necrosis). In the present study, apoptosis was exhibited in ARPE-19 cells challenged by H_2_O_2_ as demonstrated by a high-throughput flow cytometry-based method^29^. Annexin V-fluorescein isothiocyanate (V-FITC) and propidium iodide (PI) staining were analyzed by flow cytometry. As shown in Fig. 6a,b, the apoptosis rate of ARPE-19 cells markedly increased with the escalation of H_2_O_2_ concentrations and the prolongation of the treatment time. In particular, when the treatment time was increased to 24 h, the cell apoptosis rate induced by H_2_O_2_ significantly increased, indicating that the effect of prolonged treatment time on the apoptosis rate of ARPE-19 cells was greater than that of the H_2_O_2_ concentration. Notably, the apoptosis rate of ARPE-19 cells is nearest to 50% when the treatment time is 24 h and the concentration of H2O2 is 200 μM. When combined with the MTT and CCK8 analysis results, we can conclude that when ARPE-19 cells are treated with 200 μM H_2_O_2_ for 24 h, the most majority of the cell growth inhibition is due to apoptosis.

### STS inhibits apoptosis in ARPE-19 cells challenged by H_2_O_2_

**Figure 7:**
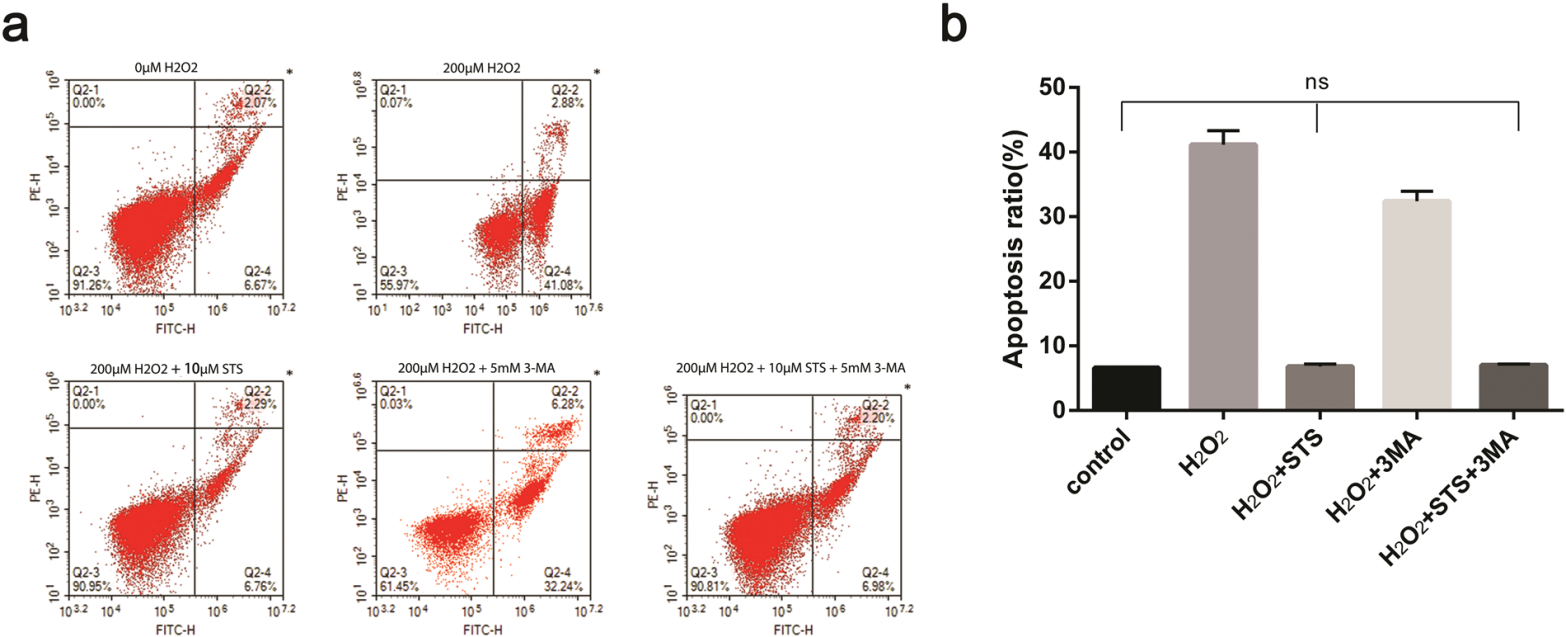
STS treatment decreases apoptosis in ARPE-19 cells under oxidative stress. (**a**) Quantification of the cell death rate by flow cytometry using V-FITC/PI labelling in each group taken by flow cytometry images. Untreated cells were used as a positive control. (**b**) The accompanied histogram of V-FITC/PI measurements of apoptotic cells. The experiment was repeated 3 times in each group. Data were analyzed using one-way ANOVA and differences between means were considered statistically significant when P < 0.05, Bonferroni correction of P-value was applied in multiple comparison. There was no statistical difference between control, H_2_O_2_ + STS and H_2_O_2_ + 3MA in (**b**), the other groups showed statistically significant differences.

We next investigated whether STS could inhibit ARPE-19 cell apoptosis under oxidative stress using flow cytometry with V-FITC/ PI labeling. As shown in Fig. 7a,b, a drastic increase of the apoptotic cell ratio was exhibited in ARPE-19 cells exposed to H_2_O_2_ compared to untreated cells. However, a remarkable reduction in the apoptotic cell ratio was shown in ARPE-19 cells treated with STS intervention under oxidative stress. Treatment with 3MA alone also resulted in a markedly decreased apoptosis rate in ARPE-19 cells in the oxidative stress environment, indicating that the inhibition of autophagy can result in the alleviation of apoptosis in an oxidative stress circumstances. Co-treatment with STS and 3MA also reduced the apoptotic rate of ARPE-19 cells, but the difference between the groups was not statistically significant in comparison with the untreated and STS only-treated cells, indicating that the STS plays a prominent role in apoptosis inhibition in ARPE-19 cells under oxidative stress.

### Effect of STS on intrinsic apoptotic pathway-related factors in ARPE-19 cells challenged by H_2_O_2_

The apoptotic signaling cascade includes two main pathways, intrinsic and extrinsic, which are triggered by different mitochondrial stimuli or by molecules binding to the cell-membrane receptor^30^. Intrinsic apoptosis signaling is induced by various stimuli, such as hypoxia, DNA damage, and oxidative stress^31^. We first investigated the mechanism of STS inhibition of ARPE-19 cell apoptosis under oxidative stress conditions from the intrinsic apoptotic pathway.

**Figure 8:**
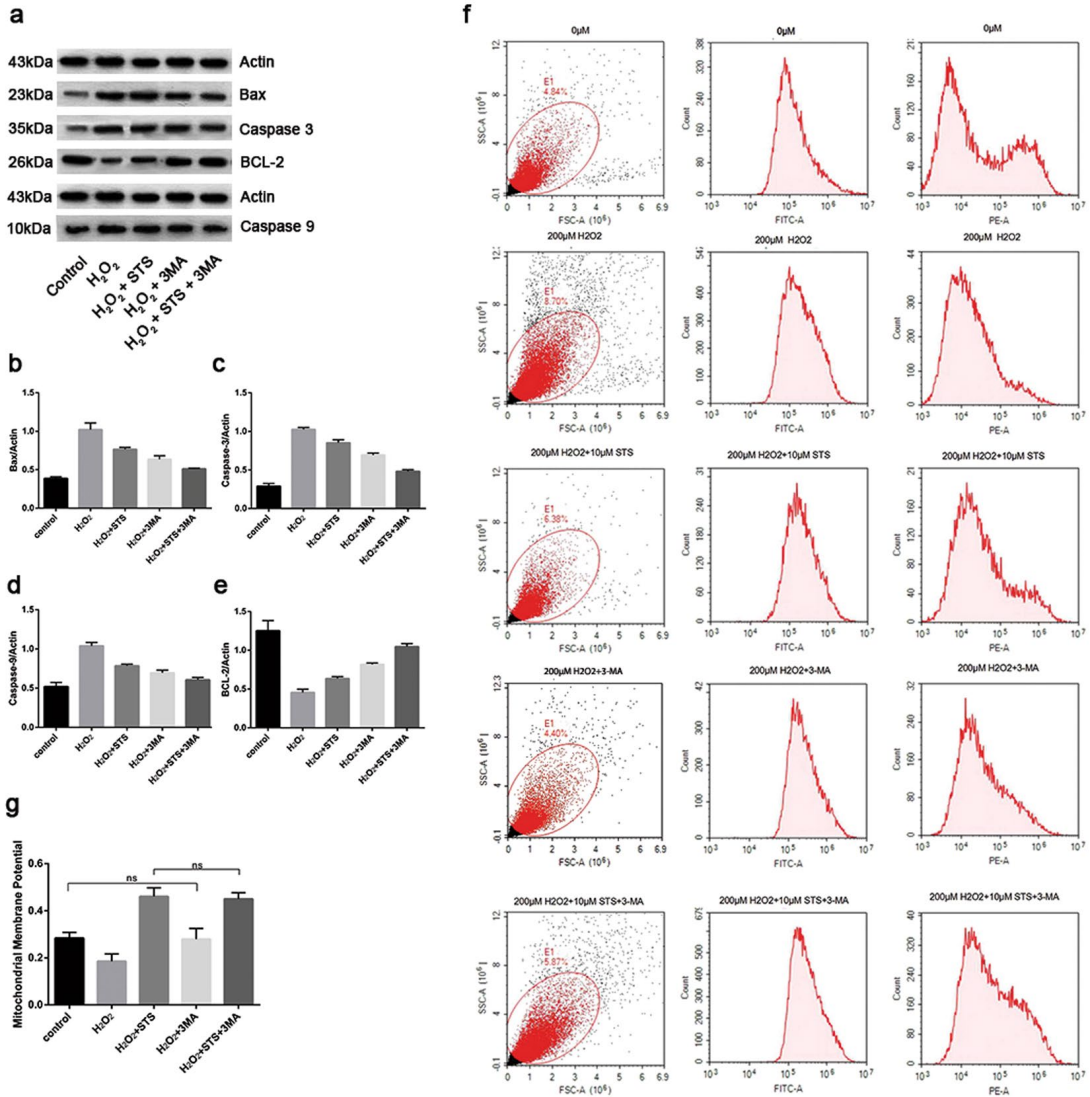
Effect of STS on intrinsic apoptosis-related factors in ARPE-19 cells under oxidative stress. (**a**) The representative protein gel blots of BAX, caspase-9, caspase-3 and BCL-2 are shown. Actin was utilized as a loading control. (**b**–**e**) The accompanying histogram of densitometric quantification of BAX, caspase-9, caspase-3 and BCL-2. Untreated cells were used as a positive control. (**f**) The effect of STS on MMP using the JC-1 dye in ARPE-19 cells under oxidative stress. The red/green fluorescent intensity ratio reflects MMP. (**g**) The accompanied histogram of MMP. The experiment was repeated 3 times in each group. Data were analyzed using one-way ANOVA and differences between measures were statistically significant when P < 0.05, Bonferroni correction of P-value was applied in multiple comparison. The statistical results show that there is no statistical difference between control and H_2_O_2_ + 3MA, or H_2_O_2_ + STS and H_2_O_2_ + STS + 3MA in (**g**), the other groups showed statistically significant differences.

The BCL-2 protein family regulates the intrinsic apoptotic pathway by controlling the mitochondrial outer membrane (MOM) integrity^32^. BCL-2 proteins are divided into two subcategories: pro-apoptotic and anti-apoptotic. Pro-apoptotic family members include BAK, BAX, BID, BAD, Noxa and PUMA. The anti-apoptotic family members include BCL-2, BCL-XL, MCL-1, BCL-W and A1/Bfl-1^33^. As shown in Fig. 8a,b and Supplementary Fig. S4, the results of WB revealed that H_2_O_2_ treatment significantly increased the expression of BAX in ARPE-19 cells compared with untreated cells. STS intervention, however, dramatically decreased the expression of BAX under oxidative stress. Concurrently, 3MA treatment alone also dampened this increase, indicating that inhibition of autophagy in ARPE-19 cells also reduced BAX expression under oxidative stress.

A reduction in the mitochondrial membrane potential (MMP) is frequently observed in cells under oxidative stress^34^. Next, we sought to demonstrate whether the STS intervention could protect APRE-19 cells from MMP loss under oxidative stress. As shown in Fig. 8f,g, H_2_O_2_ treatment markedly increased the MMP in ARPE-19 cells relative to untreated cells, indicating that oxidative stress has a destructive effect on the MOM in cells. In contrast, STS treatment caused a significant elevation in MMP, which was significantly different in comparison to the untreated group, indicating that STS can protect the MOM in ARPE-19 cells under oxidative stress. Treatment with 3MA alone also significantly increased the MMP, indicating that inhibition of autophagy also has some protective effect on the MOM in ARPE-19 cells under oxidative stress. There was no significant difference in the MMP in cells co-treated with STS and 3MA compared with cells treated with STS alone, indicating that STS plays a pivotal role in rescuing ARPE-19 cells against MMP loss compared to 3MA under oxidative stress conditions.

Caspases are cysteine proteases that are crucial to the morphological and biochemical changes that occur during apoptosis. Central events to apoptosis is the activation of caspase proteases that drive cellular disassembly^35^. As shown in Fig. 8a,c,d and Supplementary Fig. S4, the results of WB showed that the expression of caspase-9 and caspase-3 were significantly increased in ARPE-19 cells challenged by H_2_O_2_ compared with untreated cells, whereas STS intervention markedly decreased the expression of these proteins in an oxidative stress environment. Treatment with 3MA alone also reduced the expression of caspase-9 and caspase-3, confirming that inhibition of autophagy also decreased the expression of these proteins in ARPE-19 cells under oxidative stress.

BCL-2 is the primary member of the main anti-apoptotic BCL-2 family receptor proteins. As shown in Fig. 8a,e and Supplementary Fig. S4, the results of WB revealed that H_2_O_2_ treatment strongly decreased the expression of BCL-2 in ARPE-19 cells compared with untreated cells. STS intervention, however, markedly increased its expression in an oxidative stress environment. Treatment with 3MA alone also increased the expression of BCL-2, confirming that the inhibition of autophagy could reduce the expression of BCL-2 in ARPE-19 cells under oxidative stress.

### Effect of STS on extrinsic apoptotic pathway-related factors in ARPE-19 cells challenged by H_2_O_2_

**Figure 9:**
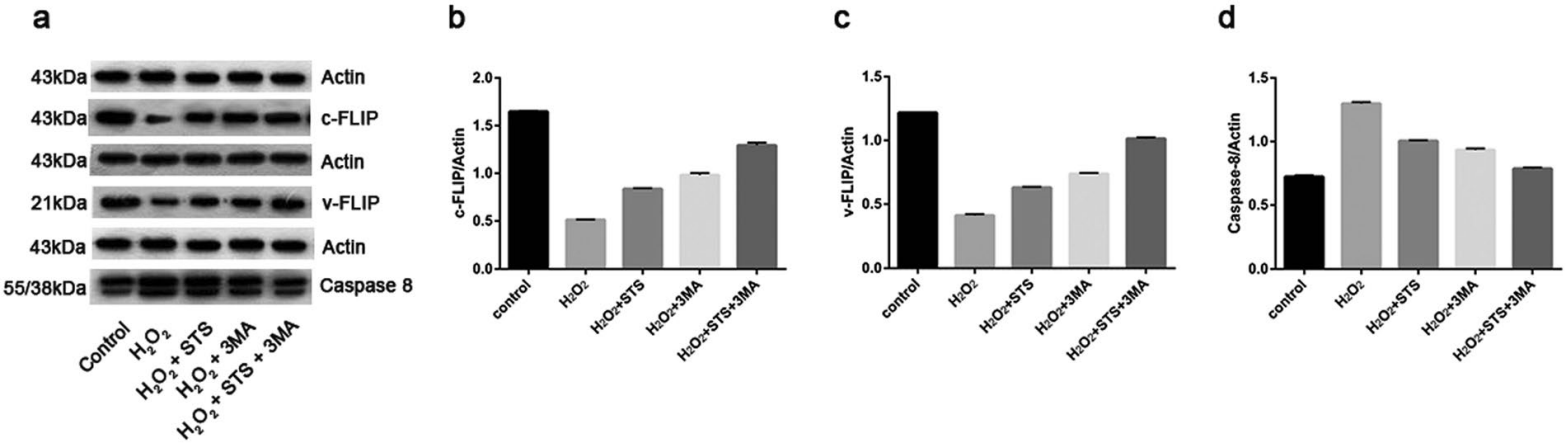
Effect of STS on extrinsic apoptosis-related factors in ARPE-19 cells under oxidative stress. (**a**) Representative protein gel blots of c-FLIP, v-FLIP and caspase-8 are shown. (**b**–**d**) The accompanied histogram of densitometric quantification of c-FLIP, v-FLIP and caspase-8. The experiments were repeated 3 times in each group. Data were analyzed using one-way ANOVA, and differences between devices were considered as statistically significant when P < 0.05, Bonferroni correction of P-value was applied in multiple comparison. The statistical results showed that there was statistical difference between the groups.

Caspase-8 is an initiator and apical activator caspase that plays a central role in apoptosis. Caspase-8 is essential for death receptor-induced activation of the extrinsic cell-death pathway^36^. Cellular FLICE-inhibitory protein (c-FLIP), a homolog of caspase-8, blocks caspase-8 apoptotic function by forming heterodimeric complexes^37^. One class of death receptor inhibitors, identified first in viruses, are the Viral FLICE-inhibitory protein (v-FLIP), which have homology with the death effect domains of caspase-8 and -10^38^. As shown in Fig. 9a–d and Supplementary Fig. S5, the results of WB showed that H_2_O_2_ treatment significantly diminished the expression of c-FLIP and v-FLIP and increased the expression of caspase-8 in ARPE-19 cells compared with untreated cells. However, STS treatment notably elevated the expression of c-FLIP and v-FLIP and decreased the expression of caspase-8 under oxidative conditions. Treatment with 3 MA alone significantly increased the expression of c-FLIP and v-FLIP and reduced the expression of caspase-8 under oxidative stress.

## Discussion

Our study demonstrates that under oxidative stress conditions: (1) Growth inhibition occurs in ARPE-19 cells, the vast majority of which is due to autophagy-associated cell death and apoptosis; (2) STS inhibits ARPE-19 cell autophagy by activating the PI3K-AKT-mTOR pathway; and (3) STS protects ARPE-19 cells from apoptosis by dampening intrinsic and extrinsic apoptosis pathways.

Recent studies have indicated the potential role of autophagy in lipofuscin accumulation, drusen formation, and AMD pathogenesis^39^. In addition, it has been reported that autophagic flux increases during aging due to the ROS-induced intracellular burden of damaged organelles and accumulated macromolecules^40^. It has been shown that autophagy plays an important role in the protection of the RPE against oxidative stress and lipofuscin accumulation, and that impairment of autophagy is likely to exacerbate oxidative stress and contribute to the pathogenesis of AMD^41^. However, autophagy itself also inevitably causes autophagic cell death. Observations that, in many instances dying cells have increased autophagic markers and morphological features of autophagy originally led to the proposal of autophagic cell death^42^. Some studies have shown that the autophagy inhibitor 3MA protects against the autophagic cell death induced by oxidative stress^43^.

Autophagy and apoptosis are two important cellular processes with complex and intersecting protein networks. Therefore, we investigated whether STS could protect ARPE-19 cells from oxidative stress, and we also explored its mechanism through both autophagy and the apoptosis pathway.

Our investigation demonstrated that STS intervention increases the expression of p62. As an important hallmark of autophagic flux, p62 binds to ubiquitinated proteins and facilitates degradation through autophagic clearance, since the proteasome is unable to remove large protein aggregates^44^. Reduction of p62 protein levels or interference with p62 function significantly increased cell death that was induced by the expression of mutant huntingtin^45^. In addition, p62 has been implicated as an adaptor protein to mediate autophagic clearance of insoluble protein aggregates in age-related diseases, including AMD. Previous findings have shown that p62 provides enhancing protection to RPE cells from environmental stress-induced protein misfolding and aggregation, by facilitating the Nrf2-mediated antioxidant response, which might be a potential therapeutic target against AMD^46^. Therefore, STS may decrease cell death and protect ARPE-19 cells against oxidative stress through the same mechanism.

Beclin 1, the mammalian orthologue of yeast Atg6, has a central role in autophagy, a process of programmed cell survival, which is increased during periods of cell stress and is halted during the cell cycle^47^. Beclin 1 is central in the regulation of autophagy due to its interaction with several cofactors that promote the formation of the BECN1-PIK3C3-PIK3R4 core complex to activate PIK3C3 lipid kinase activity, and induce autophagy^48^. Our investigation showed that STS decreased the expression of Beclin 1, suggesting that STS could dampen the formation of the BECN1-PIK3C3-PIK3R4 core complex in ARPE-19 cells under oxidative stress conditions. Atg3, an E2-like molecule, contributes to the conjugation of LC3 to PE. ATG7, an E1 ubiquitin activating-like protein, is involved in the covalent isopeptide linkage of ATG5 and ATG12 to contribute to the elongation of the phagophore membrane^49^. ATG9 is thought to play a role in the supply of components for the formation of the autophagosomal membrane^50^. STS decreased the autophagic expression of BECN1, Atg3, ATG7 and ATG9 in ARPE-19 cells under oxidative stress conditions and inhibited autophagy by lowering autophagy activation and autophagosome formation.

Our study shows that STS increases the expression of p62 in ARPE-19 cells under oxidative stress. As a central element signaling cell growth and enhancing protein translation, the mammalian target of rapamycin (mTOR), when inhibited, induces autophagy^47^. mTOR forms two distinct signalling complexes, mTOR complex 1 (mTORC1) and mTORC2, by binding with multiple companion proteins. Previous studies have shown that mTORC1 inhibits the autophagy-initiating UNC-5-like autophagy activating kinase (ULK) complex by phosphorylating complex components including ATG13 and ULK1/2^51^. Most importantly, p62 has been suggested to be a key regulator of nutrient sensing in this pathway with overexpression favouring more efficient activation of the mTOR pathway^52^. p62 is an integral part of the mTORC1 complex, and a previous study has revealed that p62 is required for mTORC1 compartmentalization and activation regulation of its recruitment to the lysosome^52^. Therefore, we can make a conclusion that STS also promotes the activation of mTOR, thereby inhibiting autophagy.

The PI3K/AKT/mTOR pathway is one of the most important signaling pathways in the regulation of cell survival, proliferation, and metabolic activities^53^. The pathway in comprised of three main driving molecules: PI3K3Kinase (PI3K), AKT, and mTOR. PI3K is a member of a subfamily of lipid kinases implicated in many physiological processes^54^. mTOR, an important component of this network, is a PI3K-related serine–threonine kinase, which can regulate anti-apoptotic and survival mechanisms by phosphorylating AKT^55^. Our results showed that STS activates the PI3K/AKT/mTOR signaling pathway in ARPE-19 cells under oxidative stress, thereby inhibiting autophagy. In addition, some studies have shown that oxidative stress by H_2_O_2_ inhibits mTOR-mediated phosphorylation of S6K1 and 4E-BP1, leading to apoptosis of neuronal cells^56^. Other studies have shown that inhibition of the PI3K/AKT/mTOR signaling pathway increased CVB3-induced CPE and apoptosis in HeLa cells^57^. Therefore, STS activates the PI3K/Akt/ mTOR signaling pathway under oxidative stress and may also play a role in inhibiting ARPE-19 cell apoptosis.

It has been shown that multiple risk factors for AMD are associated with changes in the physiological functions of RPE cells; for example, oxidants can induce apoptosis of RPE cells. The flow cytometry results using V-FITC/PI labelling showed that STS notably diminished the apoptotic rate of ARPE-19 cells under oxidative stress; therefore, we investigated whether STS inhibited cell apoptosis through intrinsic and extrinsic apoptotic pathways.

Our study showed that STS could dampen the intrinsic apoptosic pathway through a crosstalk and synergistic mechanism in ARPE-19 cells under oxidative stress. BAX is the effector of the BCL-2 family because upon activation, it changes conformation and, inserts into the outer mitochondrial membrane, oligomerizese, and induces mitochondrial outer membrane permeabilization (MOMP)^58^. Activation of the mitochondrial pathway mediates the release of cytochrome c that is associated with the opening of the MOM and loss of the MMP^59^. A significant reduction of pro-apoptotic protein expression of BAX and a notable increase of MMP were exhibited in ARPE-19 cells that were treated with STS, indicating that STS could diminish the mitochondrial permeability and decrease the release of cytochrome c. The release of cytochrome c (generally considered a critical decision point in apoptosis) involves the activation of the Bcl-2 family members BAX and BAK at the surface of mitochondria^58^. Thus, the reduced release of cytochrome c further alleviates the activation of BAX. Upon MOMP, cytochrome c translocates into the cytosol to initiate formation of the apoptosome complex, leading to proximity-induced auto-activation of initiator caspase-9 and, once activated, caspase-9 cleaves and activates executioner caspases, such as caspase-3 and -7^60^. Caspase-3 is a predominant effector caspase in apoptosis^59^. Both diminished protein expressions of caspase-9 and caspase-3 were expressed in ARPE-19 cells treated by STS under oxidative condition. BCL-2 is the primary member of the main anti-apoptotic BCL-2 family receptor protein. BCL-2 proteins block this pathway by interacting with BAX and BAK. They inhibit mitochondria permeabilization and cell death^61^. STS intervention increased the expression of BCL-2 under oxidative stress, thereby elevating its interaction with BAX, which prevented BAX and BAK from forming oligomers, further preventing it from penetrating MOM and reducing the release of cytochrome c.

Our results also showed that STS could diminish the extrinsic apoptotic pathway in ARPE-19 cells under oxidative stress. The extrinsic apoptotic pathway is activated by death receptors, which are cell-surface receptors that bind specific ligands and transmit apoptotic signals. Caspase-8 is an initiator and apical activator caspase that plays a central role in the extrinsic apoptotic pathway. STS treatment notably decreased the expression of caspase-8 in cells under oxidative conditions was exhibited in our study. Following the activation of caspases-8 and -10, the effector caspases-3, -6, and -7 are cleaved, leading to cellular degradation in the final stage of apoptosis^62^. Because apoptosis is central for the development and survival of the host, caspase-8 activation is tightly regulated. c-FLIP is a major antiapoptotic protein and an important cytokine and chemotherapy resistance factor that suppresses cytokine- and chemotherapy-induced apoptosis^63^. c-FLIP binds to caspase-8 and, in turn, blocks the formation of death-inducing signalling complex and subsequent activation of the caspase cascade. v-FLIP robustly blocks the Fas-mediated apoptosis-inducing signal by forming homophilic interactions with DEDs of FADD and/or caspase-8^64^. STS inhibited the protein expression of caspase-8 through increasing the express of c-FLIP and v-FLIP in cells under oxidative stress.

It is noteworthy that a crosstalk mechanism also exist in autoghapy and extrinsic apoptosis-related protein c-FLIP and v-FLIP. Previous study showed that c-FLIP and v-FLIP could suppress autophagy by preventing Atg3 from binding and processing LC3^65^. Our investigation showed that STS treatment significantly increased the expression of c-FLIP and v-FLIP in ARPE-19 cells under oxidative stress. c-FLIP attenuates autophagy by directly acting on the autophagy machinery by competing with Atg3 binding to LC3, thereby decreasing LC3 processing and inhibiting autophagosome formation^63^. v-FLIP also plays a critical role in concomitantly blocking cellular autophagy, whereby v-FLIP targets the autophagy effector Atg3 and impairs the elongation of the autophagosome membrane, thus inhibiting autophagy in a manner more potent than cellular FLIP^65^. It is reasonable that STS not only alleviated apoptosis by inhibiting the extrinsic apoptosis pathway, it also inhibited cellular autophagy by blocking Atg3 from binding and interacting with LC3 through c-FLIP and v-FLIP in ARPE-19 cells under oxidative stress.

Interestingly, Beclin 1, an autophagy protein, plays an indispensable role in the crosstalk between autophagy and apoptosis. This protein is part of a lipid kinase complex, and recent studies suggest that it plays a central role in coordinating the cytoprotective function of autophagy and in opposing the cellular death process of apoptosis^48^. It interacts with anti-apoptotic BCL-2-like proteins and has recently been found to be a BCL-2-homology-3 (BH3)-only protein^66^. The BH3 domain of Beclin 1 is bound to, and inhibited by, BCL-2 or BCL-XL, and this interaction can be disrupted by phosphorylation of BCL-2 and Beclin 1, or ubiquitination of Beclin 1^47^. Our findings demonstrated that STS intervention diminished the expression of Beclin 1 in ARPE-19 cells under oxidative stress, which in turn reduced the formation of the Beclin 1-BCL-2 complex and up-regulated the expression of BCL-2, robustly dampening apoptosis. The relationship between Beclin 1 and BCL-2/BCL-XL is quite complicated. Beclin 1 cannot neutralize the antiapoptotic function of BCL-2, which is exerted at the mitochondrial membrane^67^. In contrast, BCL-2 or BCL-XL reduces the pro-autophagic activity of Beclin 1^67^. Therefore, STS treatment increased the expression of BCL-2 in ARPE-19 cells under oxidative stress, which inhibited apoptosis and reduced the autophagy activity of Beclin 1.

The multiple routes of crosstalk between autophagy and apoptosis make them appear to have a balanced state between cell survival and cell death in response to different stresses. An increasing number of investigations have focused on the interaction of autophagy and apoptosis in the onset and development of neurodegenerative diseases. In this regard, the interesting question of the utilization of autophagic proteins to regulate apoptosis (and vice versa) requires further study. Our study shows that STS can inhibit autophagy and alleviate apoptosis. Among the mechanisms of crosstalk, STS activates the PI3K/AKT/mTOR pathway in ARPE-19 cells under oxidative stress to inhibit autophagy, thereby reducing autophagy-related cell death, decreasing the expression of Beclin 1, and increasing the expression of the anti-apoptotic protein BCL-2, accompanied by inhibition of the intrinsic apoptosis pathway. In addition, STS also inhibited cellular autophagy by blocking Atg3 from binding and interacting with LC3 through increasing the expression of anti-apoptotic protein c-FLIP and v-FLIP, accompanied by dampening the intrinsic apoptosis pathway. STS is expected to be candidate or to be used in a combination treatment for AMD (including dry and wet) due to its multiple protective mechanisms in ARPE-19 cells under oxidative stress conditions.

## Materials and Methods

### Cell culture and stimulation

ARPE-19 cell lines were purchased as frozen vials from the Shanghai Fu Heng Biotechnology Company. ARPE-19 cells were cultured in Ham’s F10 Medium containing 10% FBS at 37 °C in a humidified atmosphere containing 5% CO_2_/95% air, as previously described^68^. Primary cultures that were between the 2nd and 4th passage were used. ARPE-19 cells were grown to 70–80% confluence and maintained in basal media (Ham’s F10 Medium C 2% FBS) for up to 7 d to allow the cells to develop a polarized epithelial layer before the experiments were carried out. In the H_2_O_2_ stimulation experiment, ARPE-19 cells were seeded into 6-well plates at a concentration of 2 × 10^6^ cells/ml with DMEM basal medium. The H_2_O_2_ stimulation was carried out the following day using H_2_O_2_ diluted in DMEM basal medium. The ARPE-19 cells were challenged by a single administration of H_2_O_2_ (0 μM, 25 μM, 50 μM, 100 μM, 200 μM and 400 μM) for 6 h, 1 2 h and 24 h.

### MTT assay for cell inhibitory rate

The 3-(4, 5-dimethylthiazol-3-yl)-2,5-diphenyl tetrazolium bromide (MTT) assay was utilized to determine the cells’ inhibitory rate in accordance with the manufacturer’s protocol. MTT (Solarbio, Beijing, China) is light yellow or yellow alkaloid, and succinate dehydrogenase in living cells can reduce it to blue-purple-formazan, while the dead cells do not have this function. Therefore, the formation of crystalline formazan is proportional to the number of living cells. DMSO (Solarbio, Beijing, China) can dissolve this crystal, and the absorbance value is measured by a microplate reader at 490 nm. The OD value and the amount of formazan crystallization form a linear relationship within a certain range, and the impact of a drug on the survival rate of cells could be found.

### CCK8 assay for cell inhibitory rate

The CCK-8 (Cell-Counting Kit-8) assay was used to detect the cells’ inhibitory rate in accordance with the manufacturer’s protocol. CCK8 is widely utilized for the assay of cell proliferation and cytotoxicity based on WST-8. WST-8 is an MTT-like compound that can be reduced to orange-yellow formazan by some dehydrogenases in the mitochondria in the presence of an electron-coupled reagent. The amount of dye converted is directly proportional to the number of living cells and has been shown to correlate well with the 3H-thymidine incorporation assay commonly used for proliferation studies^69^. The absorbance value is measured by microplate reader at 490 nm.

### Calculate IC_50_ values by integrating the results of MTT and CCK8 assay

The calculation formula of IC50 is lgIC_50_ = Xm-I (P- (3-Pm-Pn)/4). Xm: lg maximum dose; I: lg (maximum dose/relative dose); P: the sum of the positive reaction rate; Pm: the maximum positive reaction rate; Pn: the minimum positive reaction rate.

### Selection and intervention experiments

Sodium tanshinone IIA sulfonate was purchased from Solarbio Company, and its purity was greater than 98%, as found by testing. It was diluted with DMEM basal medium (HyClone Laborotaries, Inc., Logan, Utah, USA) to the final use concentration. In the experiment, escalating concentrations of STS (10 μM, 20 μM and 30 μM) were added to the medium, and 10 μM STS was chosen for the subsequent serious of experiments according to the flow cytometry analysis results using V-FITC/PI Staining. In the STS-intervention studies, ARPE-19 cells were seeded into 6-well plates at a concentration of 2 × 10^6^ cells/ml. The following day, H_2_O_2_, STS and autophagy inhibitor 3MA (Selleckchem, Houston, Texas, USA) were diluted to their final use concentrations with DMEM basal medium, in accordance with the manufacturer’s instructions, and were added to the cells, alone or together for 24 h. The groups were follows: A: 0 μM H_2_O_2_, B: 200 μM H_2_O_2_, C: 200 μM H_2_O_2_ + 10 μM STS, D: 200 μM H_2_O_2_ + 5 mM 3MA, E: 200 μM H_2_O_2_ + 10 μM STS + 5 mM 3MA

### Detecting apoptosis by Flow cytometry analyses using V-FITC and PI staining

ARPE-19 cells were digested with 0.25% trypsin (Beyotime, Haimen, Jiangsu, China) and centrifuged at 1000 rpm for 5 min at 4 °C. The cells were then harvested. The trypsinization time should not be too long to prevent false positives. The cells were washed twice with pre-cooled PBS, centrifuged at 1000 rpm for 5 min at 4 °C, and 1–5 × 10^5^ cells were collected. Next, the PBS was discarded and 100 μl of 1 × binding buffer was added to resuspend the cells. The cells were mixed gently with 5 μL of V-FITC and 10 μL of PI staining solution (BestBio, Shanghai, China). The cells were incubated with the dyes for 10–15 min at room temperature in the dark. After the incubation, 400 μL of 1 × binding buffer was added to the cells, mixed and placed on ice. After 1 hour, the sample was detected by flow cytometry. The V-FITC-positive and PI-negative cells were recorded as apoptotic cells, and the percentage of apoptotic cells in each group was recorded.

### Detection of autophagic cells by acridine orange staining

As a marker of autophagy, the volume of the cellular acidic compartment was visualized by acridine orange (AO) staining^22^. AO (Solarbio, Beijing, China), a fluorochrome, has membrane permeability that can penetrate into acidic organelles, such as autophagy- lysosomes. Autophagic cells stained by AO can be observed to have red and yellow spots, known as acidic bodies, by a fluorescence microscope. The AO staining solution (1 mg/ml) is used as the storage solution, and should be diluted to the appropriate concentration (8.5~17 μg/ml) before use. Staining was allowed to proceed for 15~20 min at room temperature in the dark. The ARPE-19 cells stained by AO were observed by a fluorescence microscope (excitation filter wavelength 488 nm, blocking filter wavelength 515 nm) and images were taken.

### Western blot analysis

ARPE-19 cells were harvested and centrifuged at 2500 rpm for 10 min. One milliliter of RIPA cell lysate (containing 1 mM PMSF) was added to the cells, which were lysed on ice for 30 min. The supernatant was collected, which contained the total cellular protein. Cell lysates containing equal amounts of protein were loaded in each lane and were separated on a 12% SDS-PAGE gel. After separation, the proteins were transferred to a nitrocellulose membrane, and nonspecific binding sites were blocked by treatment with a solution of 5% non-fat dry milk. The membranes were then incubated with primary antibodies directed against LC3 I(1:1000, Abcam, Cambridge, MA, USA), LC3 II(1:2000, Abcam, Cambridge, MA, USA), p62(1:300, Bioss, Inc., Woburn, MA, USA), Beclin 1(1:1000, Abcam, Cambridge, MA, USA), ATG 3(1:500, Bioworld Technology, Inc., St. Louis Park, MN, USA), ATG 7(1:2000, Bioworld Technology, Inc., St. Louis Park, MN, USA), ATG 9(1:500, Bioworld Technology, Inc., St. Louis Park, MN, USA), PI3K(1:1000, Abcam, Cambridge, MA, USA), mTOR(1:500, Bioworld Technology, Inc., St. Louis Park, MN, USA), Bax(1:200, Santa Cruz Biotechnology, Inc, Dallas, Texas, USA), Caspase 9(1:200, Boster Biological Technology, Pleasanton, California, USA), Caspase 3(1:500, Bioworld Technology, Inc., St. Louis Park, MN, USA), Bcl-2(1:200, Santa Cruz Biotechnology, Inc, Dallas, Texas, USA), c-FLIP(1:300, Bioss, Inc., Woburn, MA, USA), v-FLIP(1:500, Bioss, Inc., Woburn, MA, USA), Caspase 8(1:500, Bioworld Technology, Inc., St. Louis Park, MN, USA) and were incubated overnight at 4 °C. The horseradish peroxidase (HRP)-conjugated secondary antibody was diluted according to the corresponding ratios, 1:10000 (Bax, Bcl-2, LC3-II, Caspase 3, Caspase 9, Caspase 8, ATG 3, ATG 7, ATG 9, Beclin 1, mTOR, PI3K), 1:5000 (p62), 1:2000 (LC3- I) and 1:20000 (c-FLIP, v-FLIP), in accordance with the instructions of the secondary antibodies, and was incubated at room temperature for 2 hours. The ECL Ultra-Sensitive Luminescence Kit was utilized for protein detection, referring to the instructions. The Beijing Kechuang Ruixin biological gel imaging and analysis system was used for film strip analysis (Image J software analysis of the grey band values).

### RT-qPCR analysis

One milliliter of TRIzol (Thermo Fisher Scientific, Waltham, MA, USA) was added to each well of cells and placed on ice for 5 minutes. The resulting lysate was transferred from each well into an EP tube, 0.2 ml chloroform was added and, the samples were shaken vigorously for 15 s, then incubated for 3 minutes at room temperature. The lysis samples were centrifuged at 12000 rpm for 15 minutes at 4 °C, and the supernatant (approximately 500 μl) was collected and added to a fresh EP tube. Five-hundred microliters of isopropanol was added to the supernatant, and the samples were gently mixed followed by incubation for 10 minutes at room temperature. The samples were then centrifuged at 12000 rpm at 4 °C for 10 minutes, and the supernatant was discarded. One milliliter of 75% ethanol (DEPC water preparation) was added to the samples, which were then centrifuged at 12000 rpm for 4 minutes at 4 °C, and the supernatant was discarded. The resulting RNA was dried for 30 minutes at room temperature. A total of 20–50 μL of DEPC water was added to the RNA to dissolve it, and the samples were incubated at 55 °C for 10 minutes before being stored at −80 °C for future use. Total RNA (1 μg), 10 μM oligo (dT) and DEPC water, to a final volume of 12 μL, were gently mixed in a 0.2 mL EP tube before centrifuging. The EP tube was heated in the PCR instrument and immediately cooled on ice for 3 min. Next, 4.0 μL 5 × reaction buffer, 2 ul of 10 mM dNTP mix, 1 μL of Ribolock^TM^ RNase inhibitor and 1 μL of RevertAid^TM^ M-MuLV reverse transcriptase were added to the tube. The reaction solution, which contained cDNA, was removed and stored at −80 °C. The cDNA was used as fluorescence quantitative template in the following reaction system: 5 μL of 2 × SYBR green mixture, 1 μL of forward primer (10 μM), 1 μL of reverse primer (10 μM), 1 μL of cDNA and 2 μL of Rnase-free water. All reactions were performed for 40 cycles with the following temperature profiles: 95 °C for 2 min, 95 °C for 5 s and 60 °C for 10 s. The sequences of the primers used were as follows: for β-actin, 5′-GGGAAATCGTGCGTGACATTAAGG -3′ and 5′- CAGGAAGGAAGGCTGGAAGAGTG -3′; for PI3K, 5′-CGAGTGGTTGGGCAATGAAA -3′ and 5′-TAGCAGCCCTGTTTACTGCT-3′, for mTOR, 5′-GTGGTGGCAGATGTGCTTAG -3′ and 5′-TTCAGAGCCACAAACAAGGC-3′. The analysis method used in this experiment is a relative quantification study, and the calculation method is 2^−△△Ct^.

### Assessment of mitochondrial membrane potential by JC-1 dye

JC-1 is an ideal fluorescent probe that is widely used to detect MMP, △Ψm. JC-1 dye accumulates in the mitochondria in a potential-dependent manner and can be used to detect cells, tissues or purified mitochondrial membrane potential. In normal mitochondria, JC-1 aggregates in the mitochondrial matrix to form polymers, which emit intense red fluorescence (Ex = 585 nm, Em = 590 nm), while the JC-1 that is present in the cytoplasm will exist in monomer form and emit green fluorescence (Ex = 514 nm, Em = 529 nm) due to the down-regulation or loss of membrane potential. JC-1 be used for qualitative detection due to the color change that directly reflects the change of MMP, and it can also be used for quantitative detection due to the degree of mitochondrial depolarization, which can be measured by the ratio of red/green fluorescence intensity. The cells were planted in 12-well plates at a density of 5 × 10^5^ cells/ml and grown in an incubator containing 5% CO_2_ at 37 °C overnight. Appropriate compounds were chosen for apoptosis induction according to the specific desired procedures. A 0.5 ml cell suspension sample was transferred to a sterile centrifuge tube and centrifuged at 400 × g for 5 min at room temperature, and the supernatant was discarded. The cells were resuspended with 0.5 ml of JC-1 working solution (Yeasen, Shanghai, China) and incubated for 15–30 min at 37 °C with 5% CO_2_, followed by centrifugation at 400 × g for 5 min at room temperature. The supernatant was then discarded. The cells were then washed two times by resuspending 2 ml of cell culture media or buffer and centrifuged at 400 × g for 5 min at room temperature, and the supernatants were discarded. Finally, the cells were resuspended with 0.5 ml of fresh medium or buffer for subsequent flow analysis. Healthy mitochondria containing red JC-1 aggregates were detected with the PE channel, and apoptotic or unhealthy cells containing green JC-1 monomer were detected with the FITC channel.

### Data analysis

The results are expressed as the mean ± SEM. The data analysis was performed using Prism 7.0 software (GraphPad Software, San Diego, CA, USA). Data were analyzed using one-way ANOVA or two-way ANOVA for multiple comparisons. Differences with P < 0.05 were considered statistically significant.

## Electronic supplementary material

Supplementary Figures

## Data Availability

The datasets generated during or analyzed during the current study are available from the corresponding author on reasonable request.
