# Supplementary material for: Sodium tanshinone IIA sulfonate protects ARPE-19 cells against oxidative stress by inhibiting autophagy and apoptosis
Source: Sci Rep. 2018 Oct 11;8:15137. doi: 10.1038/s41598-018-33552-2 (PMC6181947; doi:10.1038/s41598-018-33552-2)
Supplement: Supplementary file 1 — Supplementary Figures [file 41598_2018_33552_MOESM1_ESM.pdf]

# Supplementary Figures

## Sodium tanshinone IIA sulfonate protects ARPE-19 cells against oxidative stress by inhibiting autophagy and apoptosis

Dongmei Han[1], Xingwei Wu[2], Libin Liu[3], Wanting Shu[2], and Zhenping Huang[1*]

1 Department of Ophthalmology, Jinling Hospital, Nanjing, 210002, China. 2 Department of Ophthalmology, Shanghai General Hospital, Shanghai Jiaotong University School of Medicine, Shanghai, 200080, China. 3 The Third People's Hospital of Jingdezhen, Jingdezhen, 333000, China.

*Correspondence to:

**Zhenping Huang**, email: hzp19633@hotmail.com, address: 305 East Zhongshan Road, Nanjing, 210002, China.

Supplementary Figure S1

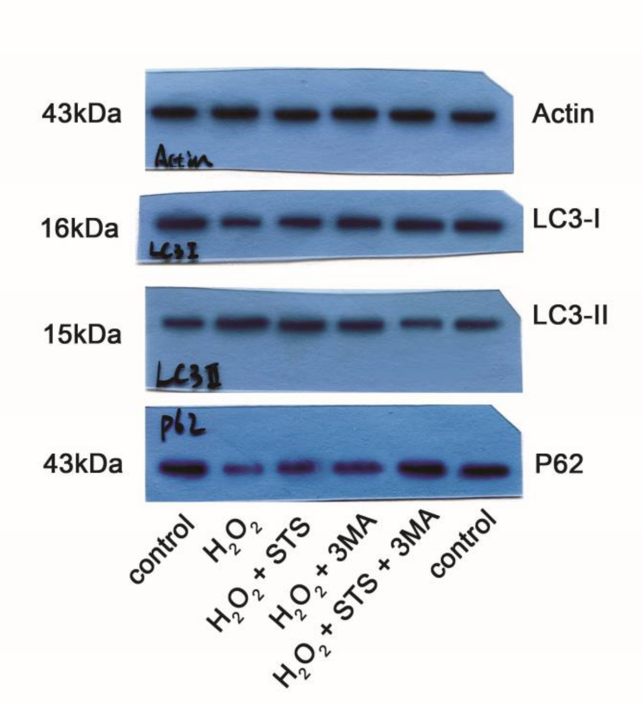


**Supplementary Figure S1.** Original blots showing LC3-I, LC3-II and p62 protein levels of ARPE-19 cells treated with STS against oxidative stress. Actin and LC3-I bands were cropped from the same gel. LC3-II and p62 bands were cropped from the same gel. In both gels, all the lanes were loaded with the same quantity of test proteins and control proteins (25μg/lane) calculated with BCA kits. Exposure time for both blots was the same (1 min).

Supplementary Figure S2

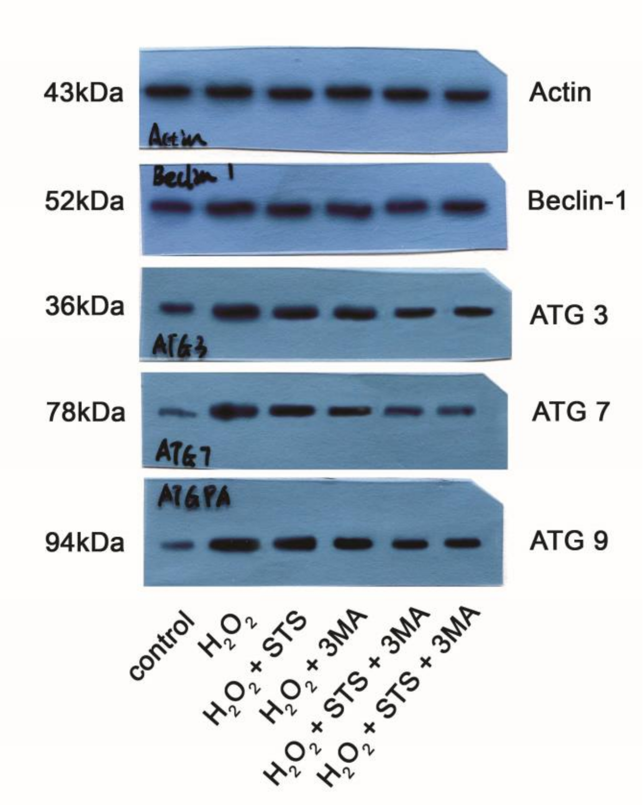


**Supplementary Figure S2.** Original blots showing BECN1, ATG3, ATG7 and ATG9 protein levels of ARPE-19 cells treated with STS against oxidative stress. Actin and ATG9 bands were cropped from the same gel. ATG3 and ATG7 bands were cropped from the same gel. In each gel, all the lanes were loaded with the same quantity of test proteins and control proteins (25μg/lane) calculated with BCA kits. Exposure time for both blots was the same (1 min).

Supplementary Figure S3

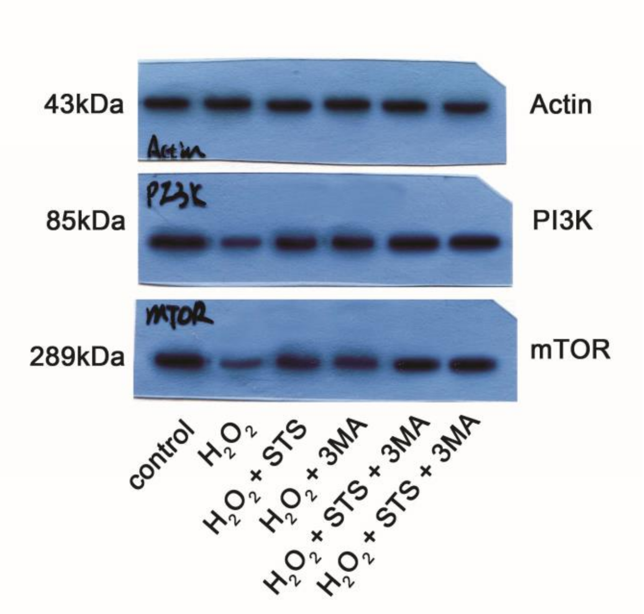


**Supplementary Figure S3.** Original blots showing PI3K and mTOR protein levels of ARPE-19 cells treated with STS against oxidative stress. Actin and PI3K bands were cropped from the same gel. In both gels, all the lanes were loaded with the same quantity of test proteins and control proteins (25μg/lane) calculated with BCA kits. Exposure time for both blots was the same (1 min).

Supplementary Figure S4

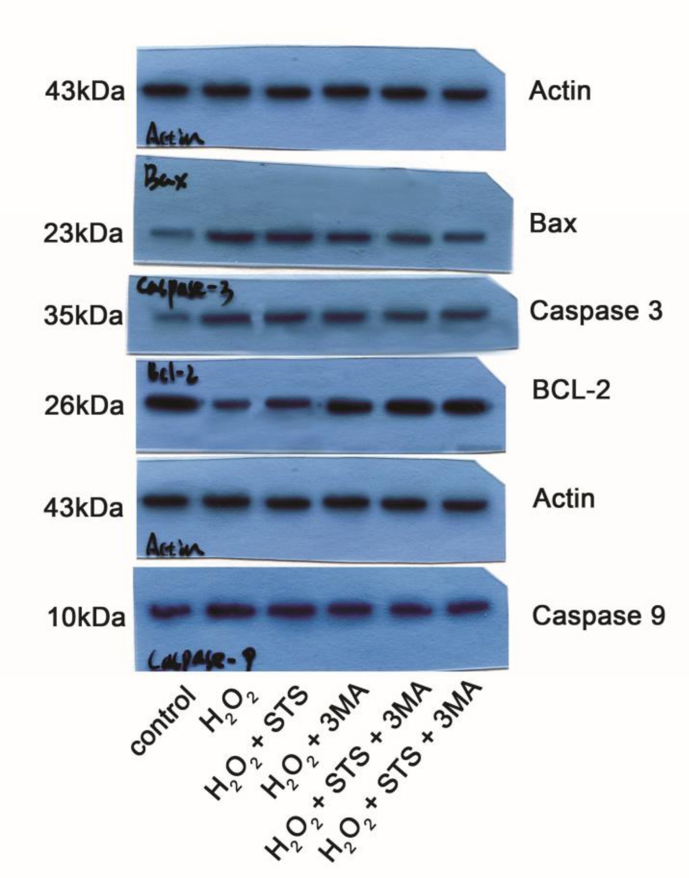


**Supplementary Figure S4.** Original blots showing BAX, caspase-3, caspase-9 and BCL-2 protein levels of ARPE-19 cells treated with STS against oxidative stress. Actin (the upper one) and Bax bands were cropped from the same gel. Actin (the lower one) and caspase-9 bands were cropped from the same gel. In each gel, all the lanes were loaded with the same quantity of test proteins and control proteins (25μg/lane) calculated with BCA kits. Exposure time for both blots was the same (1 min).

Supplementary Figure S5

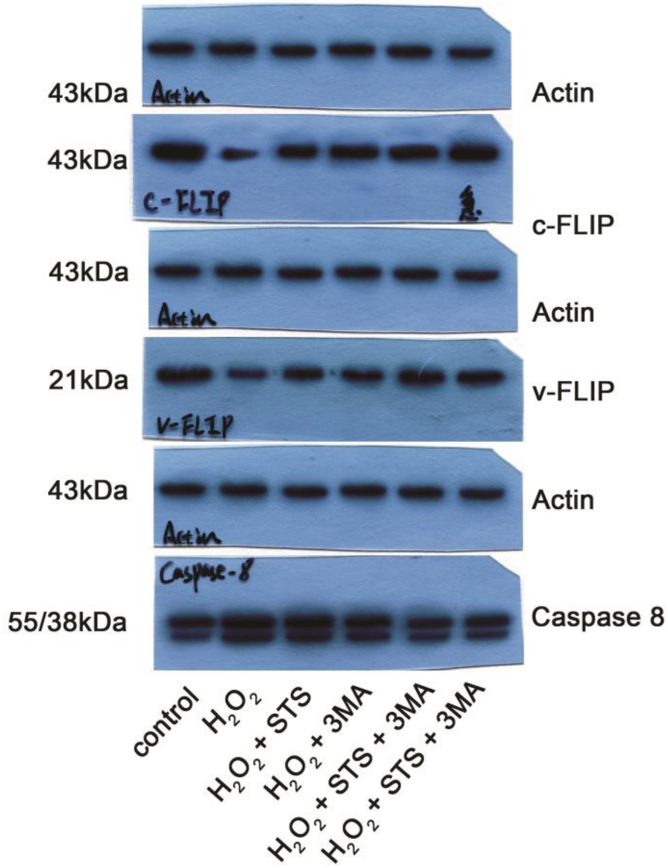


**Supplementary Figure S5.** Original blots showing c-FLIP and caspase-8 protein levels of ARPE-19 cells treated with STS against oxidative stress. Actin (the upper one) and c-FLIP bands were cropped from different gels. Actin (the middle one) and v-FLIP bands were cropped from the same gel. Actin (the lower one) and caspase-8 bands were cropped from the same gel. In each gel, all the lanes were loaded with the same quantity of test proteins and control proteins (25μg/lane) calculated with BCA kits. Exposure time for both blots was the same (1 min).
